# Retraction Note: MicroRNA-582–3p negatively regulates cell proliferation and cell cycle progression in acute myeloid leukemia by targeting cyclin B2

**DOI:** 10.1186/s11658-025-00729-3

**Published:** 2025-04-18

**Authors:** Haixia Li, Xuefei Tian, Paoqiu Wang, Mao Huang, Ronghua Xu, Tian Nie

**Affiliations:** 1 Department of Integrated Chinese and Western Medicine, Hunan Children’s Hospital, Changsha, 410007 China; 2 Hunan University of Chinese Medicine, Changsha, 410208 China; 3 College of Integrated Chinese and Western Medicine, Hunan University of Chinese Medicine, Changsha, 410208 China; 4 Department of Pediatric Rehabilitation, Hebei Provincial Hospital of Traditional Chinese Medicine, Shijiazhuang, 050000 Hebei China; 5 Department of Hematology, The First Hospital of Hunan University of Chinese Medicine, 95 Shaoshan Middle Road, Changsha City, 410007 Hunan Province China

**Retraction Note: Cellular & Molecular Biology Letters (2019) 24:66** 10.1186/s11658-019-0184-7

The Editor-in-Chief has retracted this article because of concerns regarding the figures presented in this work. These concerns call into question the article's overall scientific soundness. An investigation conducted after its publication discovered the following issues:

- the *Cyclin B2* gel slice in Fig. 4A appears to overlap with the *CDK1* gel slice in Fig. 5;
- portions of the *Cyclin B1* gel slice in Fig. 5 appear to overlap with portions of the *Bad/MCF-7* and *Bax/T-47D* gel slices in Fig. 4B in [1];
- the *miR-582-3p mimics* cell assay in Fig. 2C appears to overlap, when rotated, with the *shNek7-1* cell assay in Fig. 3E in [2].

The Editor-in-Chief therefore no longer has confidence in the integrity of the research presented in this article.

All authors agree with the retraction.

Xuefei Tian, who is listed as a co-author of this article, has stated that they did not participate in the research reported therein and that their name and affiliation were added to the author list without their consent.
